# Soluble β-amyloid Precursor Protein Alpha Binds to p75 Neurotrophin Receptor to Promote Neurite Outgrowth

**DOI:** 10.1371/journal.pone.0082321

**Published:** 2013-12-16

**Authors:** Noriko Hasebe, Yuki Fujita, Masaki Ueno, Kazuhiro Yoshimura, Yuji Fujino, Toshihide Yamashita

**Affiliations:** 1 Department of Molecular Neuroscience, Graduate School of Medicine, Osaka University, Osaka, Japan; 2 Department of Anesthesiology and Critical Care Medicine, Graduate School of Medicine, Osaka University, Osaka, Japan; 3 Core Research for Evolutional Science and Technology (CREST), Japan Science and Technology Agency (JST), Tokyo, Japan; 4 Division of Developmental Biology, Cincinnati Children's Hospital Medical Center, Cincinnati, Ohio, United States of America; 5 Department of Neurosurgery, Graduate School of Medicine, Osaka University, Osaka, Japan; National Center for Geriatrics and Gerontology, Japan

## Abstract

**Background:**

The cleavage of β-amyloid precursor protein (APP) generates multiple proteins: Soluble β-amyloid Precursor Protein Alpha (sAPPα), sAPPβ, and amyloid β (Aβ). Previous studies have shown that sAPPα and sAPPβ possess neurotrophic properties, whereas Aβ is neurotoxic. However, the underlying mechanism of the opposing effects of APP fragments remains poorly understood. In this study, we have investigated the mechanism of sAPPα-mediated neurotrophic effects. sAPPα and sAPPβ interact with p75 neurotrophin receptor (p75^NTR^), and sAPPα promotes neurite outgrowth.

**Methods and Findings:**

First, we investigated whether APP fragments interact with p75^NTR^, because full-length APP and Aβ have been shown to interact with p75^NTR^ in vitro. Both sAPPα and sAPPβ were co-immunoprecipitated with p75^NTR^ and co-localized with p75^NTR^ on COS-7 cells. The binding affinity of sAPPα and sAPPβ for p75^NTR^ was confirmed by enzyme-linked immunosorbent assay (ELISA). Next, we investigated the effect of sAPPα on neurite outgrowth in mouse cortical neurons. Neurite outgrowth was promoted by sAPPα, but sAPPα was uneffective in a knockdown of p75^NTR^.

**Conclusion:**

We conclude that p75^NTR^ is the receptor for sAPPα to mediate neurotrophic effects.

## Introduction

APP, a single transmembrane protein with a long N-terminal extracellular domain and a short cytoplasmic domain, can be processed by two distinct pathways to generate multiple cleaved products [1]. In the primary pathway, α-secretase catalyzes the cleavage of APP to generate a soluble peptide, sAPPα, which includes Aβ sequence, thereby preventing Aβ generation. In the alternative pathway, β-secretase cleaves APP to generate an alternate soluble peptide, sAPPβ, followed by γ-secretase to generate Aβ.

The start of APP expression occurs when neurons initiate differentiation at embryonic day (E) 9.5 in the mouse brain [2]. In addition, APP cleavage occurs at the embryonic stage [3]–[6] as well as injured brain tissue [7]–[9]. These observations suggest that APP fragments may have multiple roles in normal brain development and CNS injury. Indeed, it has been shown that sAPPα possesses neurotrophic effects; for example, it promotes neurite outgrowth *in vitro* [10] and protects neural tissue after brain injury [8], [11]–[13]. However, the underlying mechanism of its neurotrophic effect remains largely unknown.

p75^NTR^ mediates a diverse set of functions, including axonal elongation, neuronal survival, and modulation of synaptic transmission [14]. Furthermore, p75^NTR^ can transmit both positive and negative signals for neuronal action. For example, p75^NTR^ mediates axonal elongation through binding to neurotrophins, whereas it is also involved in axon growth inhibition through its interactions with the Nogo receptor (NgR) and LINGO co-receptors [14], [15]. Regarding APP, p75^NTR^ has been reported to associate with both full-length APP and Aβ[16]–[18]. Indeed, Aβ induces cell death via p75^NTR^ in various types of cells, including neurons [19]. This neurotoxic effect occurs through c-Jun kinase (JNK) and c-Jun [20]–[22]. A recent report further demonstrated that the N-terminal fragment of APP (N-APP) interacts with p75^NTR^ [18].

In this study, we assessed whether sAPPα and sAPPβ will also associate with p75^NTR^. We show that sAPPα and sAPPβ bind to p75^NTR^, and that sAPPα binding stimulates neurite outgrowth. These results indicate that p75^NTR^ is the receptor for sAPPα to mediate neurotrophic effects.

## Materials and Methods

### Mice

All experiments were conducted in accordance with the Osaka University Medical School Guide for the Care and Use of Laboratory Animals, and were approved by the institutional committee of Osaka University (Permit Number: 24-067-005). C57BL/6J mice were purchased from Kiwa Animal Farm (Wakayama, Japan).

### Plasmid constructs and small interfering RNA (siRNA)

Mouse sAPPα cDNA was generated by polymerase chain reaction (PCR) using primers constructed from APP valiant 2 (accession No. NM_007471) from a postnatal day (P) 4 mouse spinal cord cDNA library. The cDNA of sAPPα was inserted into a pMD20-T vector (TaKara, Shiga, Japan), and then subcloned into a pcDNA5/FRT vector (Invitrogen, Carlsbad, CA, USA). Amino-terminally Hemagglutinin (HA)-tagged full-length human p75^NTR^ was subcloned into the pcDNA3 vector (Invitrogen) [23]. Mouse p75^NTR^ siRNA was designed as described previously [24]. Scrambled siRNA was used as a negative control.

### ELISA

ELISA was performed using 96-well microplates (Thermo Fisher Scientific, Waltham, MA, USA) coated with 1% bovine serum albumin (BSA)/phosphate-buffered saline (PBS). Recombinant sAPPα (S9564, Sigma, St. Louis, MO, USA), sAPPβ (SIG-39938, Covance, Princeton, NJ, USA), or C-sAPPα (sAPPα 304–612; S8065, Sigma)–all at 12.2 nM final concentration in a final volume of 50 µL/well–was plated and incubated at 4°C overnight. After washing with PBS recombinant p75^NTR^ extracellular domain fused to human Fc (p75^NTR^ ECD-Fc) chimera protein (1157-NR, R&D Systems, Minneapolis, MN, USA) or Fc-tagged IgG (IgG-Fc) chimera protein (110-HG, R&D Systems) as a control was added to the plate at the indicated concentrations, and incubated for 2 h at room temperature. After incubation, the plate was washed, and goat anti-human IgG-Fc antibody (1∶1000; 55071, Cappel Costa Mesa, CA, USA) was added. Horseradish peroxidase (HRP)-conjugated anti-goat IgG antibody (1∶1000; sc-2020, Santa Cruz, Santa Cruz, CA, USA), substrate reagent, and stop solution (R&D Systems) were used to detect protein binding. Absorbance was measured at 450 nm.

### Pull-down assay

His-tagged sAPPα, sAPPβ, or C-sAPPα, and Ni-agarose were incubated in binding buffer (HBSS with 0.2% BSA, 0.1% NaN_3_, 5 mM CaCl_2_, 1 mM MgCl_2_, 20 mM HEPES, pH 7.0) at 4°C for 1 h. Human p75^NTR^ ECD-Fc (1157-NR, R&D Systems) or human IgG-Fc (110-HG, R&D Systems) was added to the solution, and it was incubated at 4°C overnight. Beads were washed five times with the binding buffer. Bound complexes were eluted from beads with SDS loading buffer, and subjected to sodium dodecyl sulfate polyacrylamide gel electrophoresis (SDS-PAGE, 7.5% gel), followed by western blotting with anti-sAPPα antibody (1∶50; 11088, IBL, Fujioka, Japan), anti-human p75 ECD antibody (1∶1000; AB1554, Millipore, Billerica, MA, USA) and anti-human IgG-Fc antibody (1∶1000), or anti-sAPPβ antibody (1∶500; SIG-39138, Covance).

### In situ binding of APP fragments to COS-7 cells

COS-7 cells derived from kidney fibroblast cells of monkey were cultured and maintained in Dulbecco's modified Eagle's medium (DMEM) containing 10% fetal bovine serum (FBS). The cells were plated on 3.5-cm dishes coated with poly-l-lysine (PLL) at a density of 4×10^5^ cells/mL 24 h before transfection. The cells were transfected with pcDNA3 or pcDNA3-p75^NTR^-HA by Lipofectamine 2000 (Invitrogen) according to the manufacturer's instructions. At 40 h after transfection, the cells were fixed in 4% paraformaldehyde (PFA). Non-specific binding sites were blocked in 5% BSA without detergent for 1 h. The cells were incubated with 1.22 nM sAPPα, sAPPβ, or C-sAPPα at 4°C overnight. The binding of sAPPα, sAPPβ, or C-sAPPα to p75^NTR^ was detected by immunostaining with monoclonal anti-sAPPα antibody (3∶1000) or polyclonal anti-sAPPβ antibody (1∶500), and polyclonal anti-p75^NTR^ antibody (1∶1000) with counterstaining by 4′,6′-diamidino-2-phenylindole (DAPI).

### Neurite outgrowth assay

Primary dissociated cultures of cortical neurons were prepared from E16 C57BL/6J mice by using a previously described protocol [25]. Briefly, cortices were dissected and removed, minced into small pieces on ice, and then collected in ice-cold PBS. The cells were then incubated with 0.25% trypsin (Gibco/Invitrogen, Paisley, UK) and 500 µg/mL DNase1 (Sigma) at 37°C for 15 min. Dissociated neurons were plated on 4.2-cm^2^ 2-well plastic wells coated with PLL at a density of 0.25×10^5^ neurons/dish in DMEM/Nutrient Mixture F-12 (DMEM/F12) containing B27 supplement (17504-044, Gibco) and penicillin/streptomycin (15140-122, Gibco). The neurons were incubated in the presence of human IgG-Fc or sAPPα at the indicated concentrations (1.22, 2.44, or 4.88 nM) and/or 200 nM KT5720 (420320, Calbiochem, San Diego, CA, USA) for 24 h. The neurons were then fixed in 4% PFA, and immunostained with polyclonal anti-TuJ1 antibody (1∶1000; PRB-435P, Covance). The lengths of the longest neurites were measured by ImageJ software (National Institutes of Health, Bethesda, MD, USA). Cells with neurites shorter than the diameter of its soma were excluded from the analysis.

### Nucleofection

Cortical neurons were washed and resuspended in Mouse Neuron Nucleofector Solution (Lonza, Basel, Switzerland) at a final concentration of 5×10^6^ neurons per 100 µL. The cell-nucleofector solution complex (100 µL) and the p75^NTR^ siRNA or control scrambled siRNA (500 pmol) were then gently mixed and transferred into a cuvette, followed by nucleofection using the nucleofector program O-05. Immediately after electroporation, the cells were mixed with 500 µL of pre-warmed DMEM/F12 containing 10% FBS, followed by transference of the cell suspension into 3.5-cm dishes coated with PLL. After 2 h-incubation, the medium was changed to DMEM/F12 containing B27 supplement and penicillin/streptomycin. After 3 days when the expression of p75^NTR^ was reduced by siRNA, neurons were replated on 3.5-cm dishes coated with PLL at a density of 0.5×10^5^ neurons/dish in DMEM/F12 containing 10% FBS. After another 2-h incubation, the medium was changed to DMEM/F12 containing B27 supplement, penicillin/streptomycin and 1.22 nM sAPPα or PBS control. The neurons were incubated for 24 h, fixed in 4% PFA and immunostained with polyclonal anti-TuJ1 antibody (1∶1000). The lengths of the longest neurites were measured by the ImageJ software.

### Co-culture of cortical neurons with Chinese hamster ovary (CHO) cells

CHO cells were plated on 3.5-cm dishes coated with PLL at a density of 3×10^5^ cells/dish in DMEM/F12 containing 10% FBS 24 h before transfection. pcDNA5/FRT vector or sAPPα inserted pcDNA5/FRT vector were transfected into CHO cells. The expression of sAPPα protein was confirmed as described below. At 12 h after transfection, the medium was changed to new DMEM/F12 containing 10% FBS. At 15 h after transfection, cortical neurons (0.5×10^5^ cells/dish) were plated on CHO cells. After another 2 h the medium was changed to DMEM/F12 containing B27 supplement and penicillin/streptomycin. At 40 h after co-culture, CHO cells and neurons were fixed and immunostained with polyclonal anti Tuj1-antibody (1∶1000) and monoclonal anti sAPPα-antibody (3∶1000). The lengths of the longest neurites were measured by using the ImageJ software. On the other hand expression of sAPPα was examined by western blotting. At 40 h after co-culture, CHO cells were lysed with lysis buffer (50 mM Tris-HCl, pH 7.8, 150 mM NaCl, 1% NP-40, 2 mM Na_3_VO_4_, 1 mM EDTA). The lysates and the medium of the CHO cells were centrifuged at 13,000×*g* for 5 min and the supernatants were collected. The supernatant of the CHO cell culture medium was collected and concentrated using centrifugal filter units (Amicon Ultra-0.5 mL 30 K MWCO, Millipore). The supernatant of the lysates and the concentrated supernatant of the medium were boiled in sample buffer for 5 min and subjected to SDS-PAGE. The proteins were transferred onto polyvinylidene difluoride (PVDF) membranes and blocked for 1 h in 5% skim milk. Membranes were blotted overnight with monoclonal anti-sAPPα antibody (3∶1000), followed by incubation with HRP-linked secondary antibody. For detection, an ECL chemiluminescence system (GE Healthcare, Little Chalfont, UK) was used.

### Statistical analysis

All values are expressed as mean ± SEM. Tukey-Kramer test followed by Bonferroni/Dunn test was used in growth assay by sAPPα addition. Student's t test was applied in neurite growth assay by the co-culture method. Scheffe's F test was used in neurite growth assay followed by p75^NTR^ neucleofecton. Kolmogrov-Smirnov test was applied for analysis of distribution of neurite length. P<0.05 was considered statistically significant.

## Results

### p75^NTR^ interacts with sAPPα

**Figure 1:**
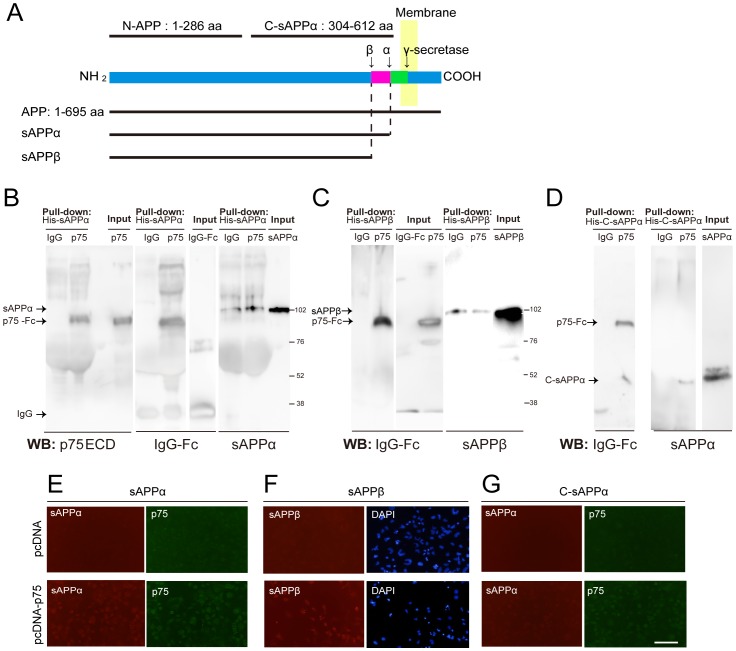
p75^NTR^ interacts with APP fragments. (A) Schematic representations of APP fragments. aa: amino acids. (B–D) Pull-down assays to assess the interaction of APP fragments with p75^NTR^. His-tagged sAPPα (B), sAPPβ (C), and C-sAPPα (D) protein were precipitated with Ni-agarose beads. p75^NTR^ ECD-Fc was co-precipitated with APP fragments. (E–G) Binding of recombinant APP fragments to p75^NTR^ on p75^NTR^-pcDNA transfected COS-7 cells. The cells were transfected with p75^NTR^ inserted plasmid or control plasmid, and the binding of sAPPα (E), sAPPβ (F), or C-sAPPα (G) on the cells was assessed by immunocytochemistry. Scale bar: 100 µm.

To assess the possible involvement of p75^NTR^ in the APP fragments (Figure 1A) signal transduction pathway, we first examined whether sAPPα interacted with p75^NTR^ by a pull-down assay. His-tagged sAPPα was incubated with Ni-agarose beads to precipitate any bound protein, and then p75-Fc or IgG-Fc as a control was added. p75^NTR^, but not control IgG protein, was detected in sAPPα precipitates (Figure 1B). Comparable experiments using sAPPβ revealed that p75^NTR^ protein was also detected in sAPPβ, and the C-sAPPα had precipitated p75^NTR^ (Figure 1C and D). C-sAPPα is the carboxyl-terminal region of sAPPα, corresponding to aa 314–612 of sAPPα (aa 1–612) (Figure 1A). These results indicate that APP fragments interact with p75^NTR^.

To examine whether APP fragments bind to p75^NTR^ on cell surfaces, we performed cell-based binding assays. COS-7 cells were transfected with either empty control vector or HA-tagged p75^NTR^ inserted vector. After 40 h, cells were fixed and incubated with recombinant protein of His-tagged sAPPα recombinant protein. Bound ligand was immunostained with anti-sAPPα antibody. sAPPα bound to cells expressing p75^NTR^ but not to cells transfected with control vector (Figure 1E). We also found that sAPPβ and C-sAPPα bound to p75^NTR^-expressing cells (Figure 1F and G). These results suggest that APP fragments bind to p75^NTR^ on cell surfaces.

### Affinity of the sAPPα-p75^NTR^ interaction

**Figure 2:**
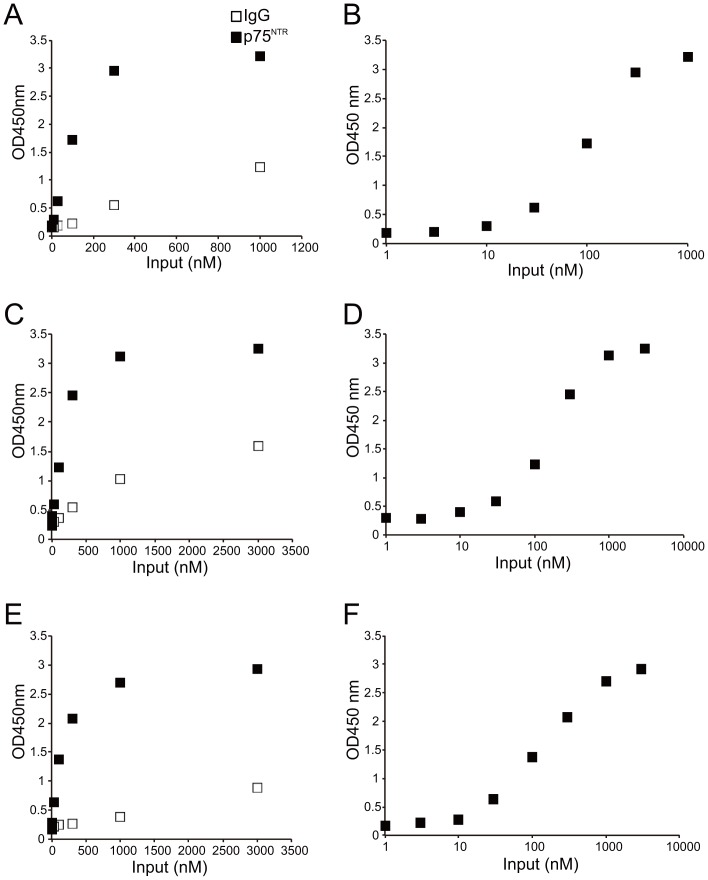
Binding affinity of APP fragments to p75^NTR^. (A, C, E) ELISA for the p75^NTR^-APP fragments interaction. sAPPα (A), sAPPβ (C), or C-sAPPα (E) was plated. After washing with PBS, p75^NTR^ ECD-Fc or IgG-Fc as a control was added to the plate at the indicated concentrations. The mean OD value after adding p75^NTR^ ECD-Fc to ELISA microwells coated with recombinant each APP peptides was higher than that of the controls. n = 3. (B, D, F) The sigmoid dose-response curve revealed the EC_50_ for each APP fragment-p75^NTR^ interaction. The EC_50_ of sAPPα (B), sAPPβ (D), and C-sAPPα (F) to p75^NTR^ were 90, 120, 150 nM, respectively.

Next, we examined the affinity of the each APP fragments-p75^NTR^ interactions by ELISA. The recombinant p75^NTR^ ECD-Fc or IgG-Fc was added to plastic wells coated with one of the APP fragments (sAPPα, sAPPβ, or C-sAPPα). The binding was detected by HRP-conjugated anti-human Fc antibody. The interaction between p75^NTR^ ECD-Fc and sAPPα was higher than that between IgG-Fc and sAPPα (Figure 2A), indicating specific binding between sAPPα and p75^NTR^ ECD. sAPPβ and C-sAPPα also bound to p75^NTR^ ECD-Fc (Figure 2C and E). The sigmoid dose-response formulas were used to calculate the EC_50_. sAPPα, sAPPβ, and C-sAPPα bound to p75^NTR^ ECD-Fc, and EC_50_ were 90, 120, and 150 nM, respectively (Figure 2B, D, F). Taken together, our observations indicate that p75^NTR^ ECD binds to APP peptides, thereby suggesting that p75^NTR^ is the receptor for APP fragments.

### sAPPα promotes neurite outgrowth

**Figure 3:**
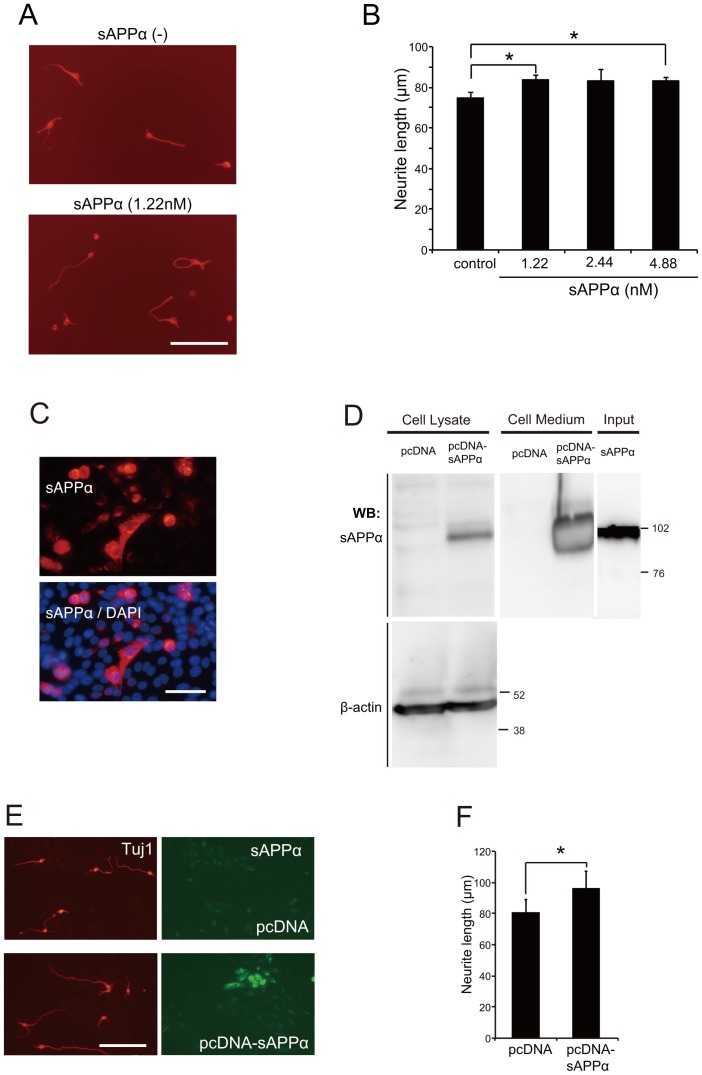
sAPPα promotes neurite outgrowth. (A, B) Cortical neurons were cultured with IgG-Fc or sAPPα at the indicated concentrations for 24 h. (A) The representative images of cortical neurons are shown. Scale bar: 100 µm. (B) The mean lengths of the longest neurite per neuron were measured by image J software and represented in the graph. The graph showed the mean ± SEM from 3 independent experiments. The number of neurons was 150 for each experiment. * *p*<0.05, Tukey-Kramer test. (C) CHO cells transfected with sAPPα-inserted plasmid were immunostained with monoclonal anti-sAPPα antibody and counterstained with DAPI. (D) sAPPα expression of CHO cells was confirmed by western blotting. The supernatants of the medium and whole cell lysates were prepared from CHO cells transfected with the indicated plasmid. Scale bar: 100 µm. (E) The representative images of cortical neurons co-cultured with transfected CHO cells are shown. Scale bar: 100 µm. Left panels display immunostaining of neurites with polyclonal anti-Tuj1 antibody. Those neurons were co-cultured with CHO cells transfected with pcDNA as a control or pcDNA-sAPPα. Right panels display immunostaining of CHO cells with sAPPα. (F) Neurite outgrowth was significantly promoted when the neurons were cultured on sAPPα-expressing CHO cells compared with the culture on control CHO cells. The mean lengths of the longest neurite per neuron were measured by image J software and represented in the graph. The graph showed the mean ± SEM from 7 independent experiments. The number of neurons was 150 for each experiment. * *p*<0.05, Student's *t*-test.

It has been reported that sAPPα exerts neuroprotective effects in the traumatic brain injury model [8], [11]–[13]. Therefore, we focused on cortical neurons to examine the effect of sAPPα on neurite outgrowth. For this purpose, we compared the neurite length of sAPPα-treated neurons and control ones. Cortical neurons from E16 mice were treated with IgG-Fc as control or sAPPα at concentrations of 1.22 nM, 2.44 nM, or 4.88 nM, and cultured for 24 h. Neurite outgrowth was enhanced by sAPPα treatment (Figure 3A, B, and S1A).

We further examined neurite length by the co-culture method. In this method, CHO cells were transfected with either empty vector or His-tagged sAPPα inserted vector. We observed that sAPPα protein expression was only detected in sAPPα-transfected CHO cells (Figure 3C and D). Neurite outgrowth was promoted when the neurons were cultured on sAPPα-expressing CHO cells, compared to those on control CHO cells (Figure 3E, F, and S1B). These results demonstrate that sAPPα promotes neurite outgrowth in embryonic cortical neurons.

### p75^NTR^ is required for sAPPα–induced neurite outgrowth

**Figure 4:**
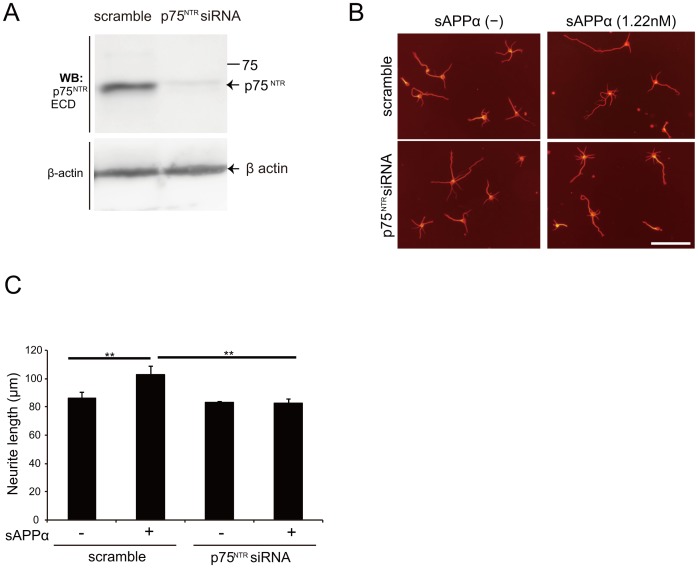
p75^NTR^ mediates sAPPα-induced neurite outgrowth. (A) p75^NTR^ siRNA specifically reduced target protein expression. Cortical neurons were transfected with scrambled control or p75^NTR^ siRNA. Cell lysates were prepared 72 h after transfection and subjected to western blotting. β-actin expression was used as an internal control. (B, C) siRNA-mediated knockdown of endogenous p75^NTR^ suppressed sAPPα-induced neurite outgrowth. (B) Representative images of cortical neurons are displayed. Cortical neurons were transfected with scramble siRNA (control) or p75^NTR^ siRNA. Three days after transfection, the neurons were incubated in the presence or absence of sAPPα for 24 h. Scale bar: 100 µm. (C) The mean lengths of the longest neurite per neuron were measured by image J software and represented in the graph. The graph showed the mean ± SEM from of three independent experiments. The number of neurons was 150 for each experiment. ** *p*<0.01, Scheffe's F test.

The aforementioned results suggest that p75^NTR^ interacted with APP fragments (Figure 1 and 2). To address whether p75^NTR^ is a functional receptor for sAPPα, we performed a series of loss-of-function experiments using siRNA for p75^NTR^ [24]. We first confirmed the knockdown efficacy of p75^NTR^ siRNA in cortical neurons endogenously expressing p75^NTR^. Efficient downregulation of p75^NTR^ protein was specifically observed in p75^NTR^ siRNA-transfected cells (Figure 4A), indicating successful siRNA-mediated knockdown of p75^NTR^ protein. We next examined whether p75^NTR^ mediated neurite elongation by sAPPα. sAPPα promoted neurite outgrowth of E16 cortical neurons up to 18.7% of control levels. Transfection of p75^NTR^ siRNA reversed the effect of sAPPα on neurite outgrowth to control levels (Figure 4B, C, and S1C). These results demonstrate that p75^NTR^ mediates the promotion of neurite outgrowth by sAPPα.

### PKA mediates sAPPα-induced neurite outgrowth

**Figure 5:**
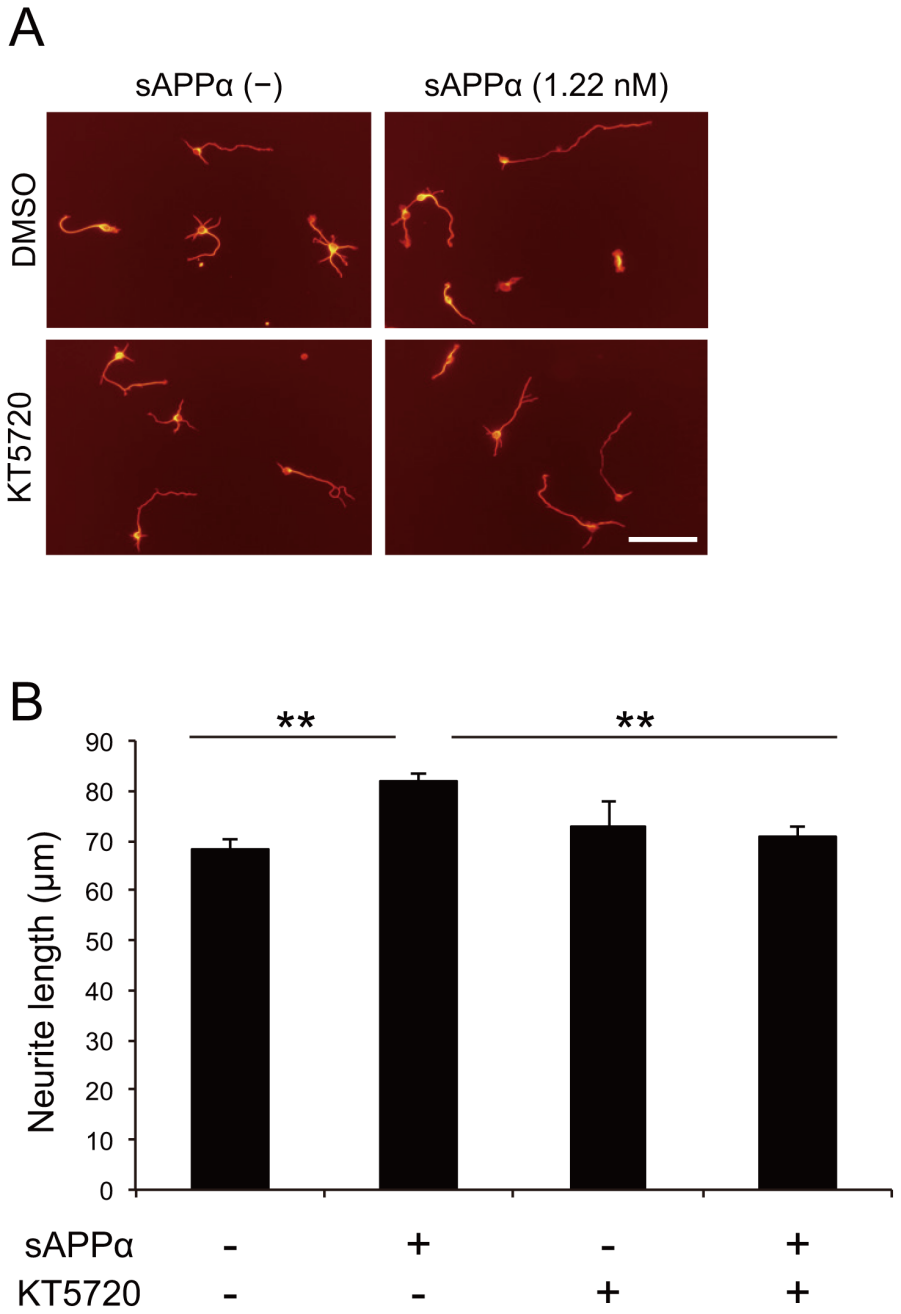
PKA is required for sAPPα-induced neurite outgrowth. (A, B) Inhibition of PKA abolished the sAPPα-induced neurite outgrowth. Neurons were cultured with sAPPα and/or PKA inhibitor, KT5720 for 24 h. (A) The representative images of cortical neurons are shown. Scale bar: 100 µm. (B) The mean lengths of the longest neurite per neuron were measured by image J software and represented in the graph. The graph showed the means ± SEM of three independent experiments. The number of neurons was 150 for each experiment. ** *p*<0.01, Tukey-Kramer test.

Our previous study demonstrated that neurotrophin binding to p75^NTR^ promoted neurite outgrowth through cyclic adenosine monophosphate-protein kinase A (cAMP-PKA) [26]. We examined the hypothesis that cAMP-PKA is located downstream of p75^NTR^ in the signaling pathway mediated by sAPPα. We confirmed that treatment of sAPPα to the culture of cortical neurons significantly enhanced neurite outgrowth in the presense of DMSO, which was used as a solvent control for PKA inhibitor KT5720. By contrast, treatment with KT5720 suppressed the effect of sAPPα on neurite outgrowth (Figure 5A, B, and S1D). These results demonstrate that PKA activation is essential for sAPPα-induced neurite outgrowth.

## Discussion

In this study, we demonstrated that sAPPα binds to p75^NTR^ and promotes neurite outgrowth. Furthermore, sAPPβ also binds to p75^NTR^. These results implicate p75^NTR^ as the receptor for sAPPα in promoting neurite outgrowth.

Although the effect was modest, sAPPα significantly enhanced the neurite outgrowth (Figure 3A and B). We observed that the treatment of sAPPα increased the number of cells, which had neurites longer than 180 µm (Figure S1A). These results demonstrated modest but significant effects of sAPPα on neurite elongation. In some cases, excessive neurite elongation may burden on the cells. To promote axon outgrowth, neurons undergo expansion of the plasma membrane [27]. Therefore, rapid neurite outgrowth may result in exhausting cellular biosynthesis. It is possible that sAPPα promotes neurite outgrowth with a lower stress on neurons.

We calculated the EC_50_ of the p75^NTR^-APP fragments interaction with ELISA. The EC_50_ of sAPPα-, sAPPβ-, and C-sAPPα–p75^NTR^ interactions were 90, 120, and 150 nM, respectively (Figure 2B, 2D and 2F). It was previously reported that the EC_50_ of the N-APP (APP 1–286)-p75^NTR^ interaction is 300 nM [18]. C-sAPPα (304–612) and sAPPα (1–612) share the common region 304–612 aa (Figure 1A). C-sAPPα binds to p75^NTR^, whereas N-APP (1–286) also binds to p75^NTR^ [18]. These findings indicate that sAPPα binds to p75^NTR^ in both N- and C-terminal regions of sAPPα. That is why sAPPα bind to p75^NTR^ with greater affinity by binding both regions.

In addition, both fragments possess the region involved in the promotion of neurite outgrowth [28]–[31]. These observations suggest that both N- and C-terminal regions of sAPPα contribute to interaction with p75^NTR^ and the regulation of neurite outgrowth.

We observed that the EC_50_ of sAPPα-p75^NTR^ interaction was lower than that of sAPPβ–p75^NTR^, indicating that, while sAPPβ also binds to p75^NTR^, sAPPα binds to p75^NTR^ with greater affinity. It was reported that sAPPα is more efficient in protecting hippocampal neurons and promoting neurite outgrowth compared to sAPPβ [32], [33]. These findings suggest that the greater binding affinity of sAPPα- p75^NTR^ might affect the neuroprotective and neurotrophic function of sAPPα.

Previous study suggested that sAPPs possibly modulated NGF–p75 signaling pathway [34]–[37]. In this study, we demonstrated that p75^NTR^ knockdown blocked sAPPα-induced neurite elongation, suggesting the involvement of p75^NTR^ in sAPPα-induced neurite outgrowth. Moreover, we revealed that sAPPα promotes neurite outgrowth through the PKA signaling pathway. p75^NTR^ mediates neurite elongation via cAMP-PKA signaling pathway [26]. Nerve growth factor (NGF) promotes neurite outgrowth in embryonic rat hippocampal neurons and chick ciliary neurons [38]. Binding of NGF to p75^NTR^ activates cAMP-PKA, and translocates p75^NTR^ to lipid rafts, resulting in neurite outgrowth [26]. Therefore, it would be reasonable to implicate cAMP-PKA involvement in the downstream signaling mediated by sAPPα-p75^NTR^.

In contrast, p75^NTR^ also functions as a signal transducer of neurite outgrowth inhibition. When myelin-derived proteins bind to the NgR, which lacks an intracellular domain, p75^NTR^ interacts with NgR to transduce the inhibitory signals intracellularly [39]. Next, p75^NTR^ facilitates the release of RhoA from Rho-GDP–dissociation inhibitor (Rho-GDI), resulting in RhoA activation. The activation of RhoA has a critical role in inducing the inhibition of neurite outgrowth [40]. In this study, we showed that the PKA inhibitor KT5720 inhibited sAPPα-induced neurite outgrowth. These observations lead to our hypothesis that sAPPα also suppresses RhoA activation through p75^NTR^. Further studies are required to assess the validity of this hypothesis.

Additionally, APP cleavage occurs during embryogenesis [3]–[5], suggesting that APP fragments are required for embryonic development. In addition, the axons of p75 mutant embryos are disturbed [41]. Based on these findings, sAPPα-p75^NTR^ signaling may be involved in normal brain development. Furthermore, APP is expressed and cleaved dramatically in CNS injuries, such as spinal cord or traumatic brain injuries [7]–[9]. Therefore, APP cleaved products and the p75^NTR^ signal may affect the recovery process of neural tissues. Understanding the molecular pathway may assist in the elucidation of novel therapeutic targets for CNS diseases.

In conclusion, we revealed that both sAPPα and sAPPβ interact with p75^NTR^ on COS cells. Knockdown of p75^NTR^ suppressed the effect of sAPPα. These results support the hypothesis that p75^NTR^ is the receptor for sAPPα in neurite outgrowth.

## Supporting Information

Figure S1 **Distribution histograms of the neurite length.** (A) Cells were treated with human IgG-Fc or various doses of sAPPα for 24 h. sAPPα increased the ratio of the longer neurites. n = 3. (B) Cortical neurons were cocultured with mock or sAPPα-transfected CHO cells for 24 h. Neurons cocultured with sAPPα-transfected CHO cells increased the ratio of the longer neurites. n = 7. (C) Cortical neurons were transfected with scramble siRNA (control) or p75 siRNA. Three days after transfection, the neurons were incubated with sAPPα for 24 h. Knockdown of p75^NTR^ reversed the effect of sAPPα on longer axons to control levels. n = 3. (D) Cortical neurons were treated with sAPPα for 24 h and/or PKA inhibitor, KT5720. Treatment with KT5720 suppressed the effect of sAPPα on neurite outgrowth. n = 3. The mean lengths of the longest neurite per neuron were measured by image J software and represented in the graph. The graph showed the mean ± SEM of independent experiments. The number of neurons was 150 for each experiment. ** *p*<0.01, Kolmogorov-Smirnov test.
(TIF)
