# Supplementary figures and images for: Soluble β-amyloid Precursor Protein Alpha Binds to p75 Neurotrophin Receptor to Promote Neurite Outgrowth
Source: PLoS One. 2013 Dec 16;8(12):e82321. doi: 10.1371/journal.pone.0082321 (PMC3864954; doi:10.1371/journal.pone.0082321)

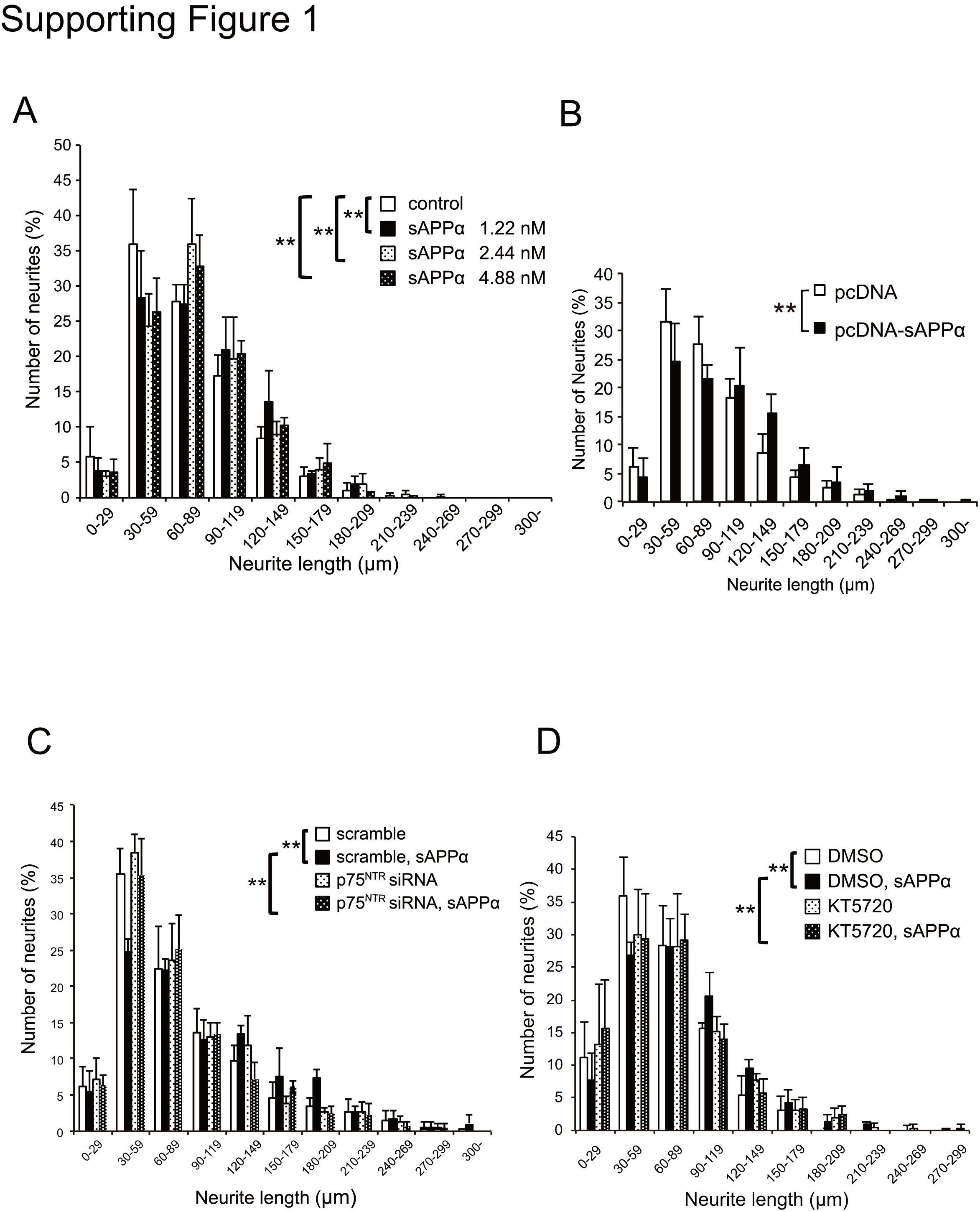

Supplement: Figure S1 — Distribution histograms of the neurite length. (A) Cells were treated with human IgG-Fc or various doses of sAPPα for 24 h. sAPPα increased the ratio of the longer neurites. n = 3. (B) Cortical neurons were cocultured with mock or sAPPα-transfected CHO cells for 24 h. Neurons cocultured with sAPPα-transfected CHO cells increased the ratio of the longer neurites. n = 7. (C) Cortical neurons were transfected with scramble siRNA (control) or p75 siRNA. Three days after transfection, the neurons were incubated with sAPPα for 24 h. Knockdown of p75NTR reversed the effect of sAPPα on longer axons to control levels. n = 3. (D) Cortical neurons were treated with sAPPα for 24 h and/or PKA inhibitor, KT5720. Treatment with KT5720 suppressed the effect of sAPPα on neurite outgrowth. n = 3. The mean lengths of the longest neurite per neuron were measured by image J software and represented in the graph. The graph showed the mean ± SEM of independent experiments. The number of neurons was 150 for each experiment. ** p<0.01, Kolmogorov-Smirnov test. (TIF) [file pone.0082321.s001.tif]
